# Medical News and Items

**Published:** 1877-04

**Authors:** 


### Medical News and Items.

Sir William Fergusson, the distinguished English surgeon, died February 10th.

---

Dr. Gurdon Buck, well-known especially for his achievements in autoplastic surgery, died at New York, March 6th.

---

With the deepest regret we inform our readers that Prof. J. W. Freer, President of the Faculty of Rush Medical College, has been seriously ill the past two weeks, and is now in so precarious a state, as to give rise to the greatest anxiety.

---

Two new journals have reached us. *The Toledo Medical and Surgical Journal* and *The Quarterly Journal of Inebriety.* The former is a monthly, and Dr. Jonathan Priest is the editor. The latter is the official organ of the American Association for the Cure of Inebriates, and the first number does the projectors credit. It is under the management of Dr. T. D. Crothers, Binghampton, N. Y.

---

At the New York Ophthalmic and Aural Institute 4,709 patients were treated during the past year; 3,873 for diseases of the eye, and 836 for diseases of the ear. From the time this charity was opened, May, 1869, to the 31st of December, 1876, 28,191 patients have been treated in the dispensary, and 2,205 admitted to the house; and 3,130 operations have been performed during that time.

The Central Free Dispensary of West Chicago received, a few days ago, a generous donation of twelve cases of extract of malt with iron, cod liver oil, and pepsine, from Tromers Extract of Malt Company.

---

The *Alumni of the County Hospital* held their annual re-union, March 21st, in the amphitheatre of the new hospital. About twenty gentlemen attended this pleasant meeting, to renew the old acquaintance with the air and diet of the hospital.

---

At the last quarterly meeting of the Michigan State Board of Health, Prof. R. C. Kedzie read an interesting report on the quality of illuminating oils in use in Michigan. The professor, by the advice of the Board, had visited Cleveland in order to examine the methods of refining the crude petroleum to make illuminating oils. His investigations established the following facts: That to manufacture good oil which will stand the test required by the Michigan law for inspection, requires either extra care and expense in refining, or it takes what is called the "heart of the run." This is done, in the case of what is known as "Water White Oil," which is transparent, burns with a clear bright light, and does not become opalescent by exposure to a temperature of 32° F. But to dispose of the inflammable products and still furnish an oil which will stand the Michigan test, the refiners run into the low-test oil the waxy and tarry products, paraffine, etc., which are not readily combustible, and which raise the standard of the low-test oils, rendering them capable of standing the test, but of poor quality for lighting purposes. Among the specimens exhibited was four ounces of paraffine taken from one quart of oil. The presence of a large amount of paraffine has a tendency to gum up the wick and render it incapable of supplying oil to the flame. Dr. Kedzie found that the "Water White Extra" would flow through 93 millimetres of tube containing wicking, and burn freely, while the "Michigan Test,"

or the oil that contains so much paraffine would flow through only seven millimeters. The action of sunlight upon oil spoils its burning qualities. It developes in the oil a quantity of tarry matter. Two specimens of oil, drawn from the same barrel, one of which had been exposed to the light for a few weeks, and the other kept in an opaque bottle, were exhibited. The one exposed to the light was decidely yellow and clouded, while the other was still white and transparent. If the test could be extended to ascertain the amount of paraffine present, and rejecting such oil as contains it in amounts which impede the capillary action of the wick, or render the oil hard or waxy in cold weather, it might do very much towards removing the difficulties of which the people complain. The best test which he could now propose for this was: the oil should remain clear when its temperature was reduced to 32° F.

---

Library of the Medical Press Association. *A Card from the Librarian.*—The Library has been removed to its new quarters at 188 South Clark street, room 9. It will be kept open, in charge of a competent assistant, from 10 to 4 o'clock daily, except Sundays.

The *Index Rerum* of periodicals, reports and transactions, is sufficiently advanced to be very useful for purposes of reference, and its usefulness will increase month by month as it is made more complete.

Donations of books, pamphlets, and papers, are earnestly solicited.

The following rules for the government of the Library have been established by the Board of Directors:

I. No books shall be allowed to be taken from the library room.

2. The use of the library is restricted to members of the Association; to physicians who pay five dollars annually for the privilege; to visiting regular practitioners from abroad.

Norman Bridge, *Librarian.*

The eighteenth annual commencement of the *Chicago Medical College* took place at Plymouth church, March 20. Prof. N. S. Davis delivered the address to the graduates, in whose behalf G. O. Rutledge responded. The degree of M. D. was conferred upon the following gentlemen: John P. Bading, Elizur K. Bailey, Frederick A. Beck, Victor A. Bergeron, Charles D. Boardman, George W. Bothwell, James Brooks, James Brown, Justin H. Burdick. Robert A. Carson, George P. Chenoweth, Edgar V. Dales, Charles S. Dickson, Sam. F. Farrar, George F. Fleischman, Lucius F. Foote, Gustavus H. Gray, Truman A. Hand, Theodore F. Johnson, Charles D. Jones, William H. Kirby, Nathaniel S. Lane, Edwin R. Lovesee, Frederic L. Marcotte, Isaac McComb, Henry H. McGray, Frank P. Nourse, Hiram L. Pease, Joseph I. Pogue, George W. Pratt, John G. Reid, George O. Rutledge, Frank F. Safford, Frederick Schoop, Frank W. Searles, Gustavus A. H. Sienank, Edward H. Webster. Ad eundum degree upon Isaac L. Potter; honorary degree upon Julius A. Freeman.

---

Recent German medical journals contain reports of cases of pemphigus acutus neonatorum which have appeared in several of the clinics, and in private houses, particularly in the practice of midwives. This disease seemed to cling to the practice of midwives with stubborn tenacity, as is shown by a long array of facts and cases reported by Dr. Dohrn. The vesicles were in some instances few in number, but in others the erythema was considerable. In one child that died, the eruption of vesicles was so extensive that those persons who saw it declared that the appearance of the corpse was horrible, almost the entire body being covered with atheromatous crusts. In most cases no fever attended the eruption, and the general health of the children was not visibly influenced. The midwives in whose practice cases were appreciably appearing, were advised by the physicians of Wiesbaden to observe the utmost cleanliness, to use disinfecting washes, to change their clothing, to visit the well children before the sick, and to use the thermometer to carefully determine the temperature of the children's baths—and yet the disease continued to spread. Dr. Dohrn advised the midwives to quit practice for four weeks, and this effectually checked the progress of the disease, as was shown by careful statistics.

---

MEMOIR OF DR. BENJAMIN RUSH. Doctor Rush was born on a farm 13 miles from Philadelphia, on the 24th of September, 1745. At the age of 9 years he was placed under private instruction, and so remained until he was 14 years of age, at which time he was sent to Princeton College, receiving the degree of Bachelor of Arts from that institution in his 17th year. Subsequently, he took up the study of medicine under Dr. John Redman.

Having remained 6 years with Dr. R., and also having prosecuted his studies with great diligence and success; translating in the meantime, "Aphorisms of Hippocrates," from the original Greek, also, works of Bœerhaave and Sydenham from their original Latin, he was compelled in consequence of there not being, at that time, a medical college in America, to go abroad to complete his medical education. So, in 1766 he went to Edinburgh, under the tutorship of Dr. Cullen. After two years of close study in that institution, he took his medical degree. Remaining nearly a year in the London hospitals, he returned to Philadelphia, and entered at once upon busy, and successful practice. Soon, however, he was appointed to the chair of Chemistry, in the medical college of Philadelphia; (this being at that time the only medical college in America, and about two years old), and a few years later to the chair of Theory and Practice which he retained until his death in 1813. He remained in the Medical College of Philadelphia, and taught medicine 44 years.

Dr. R. was the discoverer of, and first to teach the true pathology of the dropsies. The treatment of hydrocephalus was greatly advanced by his careful clinical and autopsical studies. To Rush is due the credit of separating the American medical mind from European authority; thus securing the first impulses to independent medical thought. In 1793 an epidemic of yellow fever developed in Philadelphia, which destroyed in three months four thousand lives. Rush was one of the few who remained at their posts. The courage and fortitude with which he labored in this epidemic, his efforts to establish sanitary protection of cities—the first effort ever made—against yellow fever, spread his name far and wide. "Kings and emperors sent him tokens of appreciation; learned societies of Europe sent him testimonials and enrolled him in honorary memberships; and the most famous European physicians wrote in praise of him." As a statesman and orator, Rush excelled. He was one of the first to advocate and support American independence. As a writer he was effectual. It was he who influenced Thos. Paine to write his pamphlet entitled "Common Sense," which did more to promote the liberty and independence of the American people, than any other single publication; and of the five physicians who signed the Declaration of Independence, Rush was one.—*Detroit Med. Journal, Feb.* 1877.

